# Six areas of consideration when designing and conducting online surveys in microbiology for facilitating improved scientific communication

**DOI:** 10.3389/fmicb.2023.1288822

**Published:** 2023-12-05

**Authors:** Enriqueta Garcia-Gutierrez, Liam H. Walsh, Paul D. Cotter

**Affiliations:** ^1^Food Bioscience Department, Teagasc Food Research Centre Moorepark, County Cork, Ireland; ^2^APC Microbiome Ireland, University College Cork, County Cork, Ireland; ^3^VistaMilk SFI Research Centre, Moorepark, County Cork, Ireland; ^4^School of Microbiology, University College Cork, County Cork, Ireland

**Keywords:** scientific communication, survey, gut microbiota, microbiology, fermented foods

## 1 Introduction

Scientific communication is becoming ever more important (Burns et al., 2003). It goes beyond imparting scientific knowledge and includes a focus on enhancing public understanding of the role and impact that science can have in society. Scientific-based communication facilitates conversations with the general public or specific sectors and can empower individuals and communities to participate in scientific processes, such as citizen science, which can even give people a voice in local decision-making (Bonney et al., 2016). Moreover, it can raise awareness about how public money is used for science-based interventions to tackle societal challenges, e.g., during the COVID-19 pandemic, and contribute to the understanding of the roles of researchers (Nosek and Bar-Anan, 2012).

Unsurprisingly, public outreach activities are encouraged by the vast majority of academic institutions and funding bodies, and many programmes and activities have been developed to make science more accessible to the non-academic population (Caballe and Bardelli, 2022; Rouzer et al., 2023). Microbiology is not an exception and, as a result, there has been an increase in public dissemination actions in recent years (Chang, 2011). Surveys represent a flexible avenue for gathering information to facilitate better communication between researchers and the general public. For example, surveys can be employed to determine the general population's, or a specific group's, awareness of microbiology-related topics and identify hot themes from the scientific literature. Moreover, one of the most impactful reasons for carrying out surveys is that the results can be used to create effective communications that target gaps and misconceptions in people's mental frameworks, thereby helping to address misinformation (Bruine de Bruin and Bostrom, 2013).

**Figure 1:**
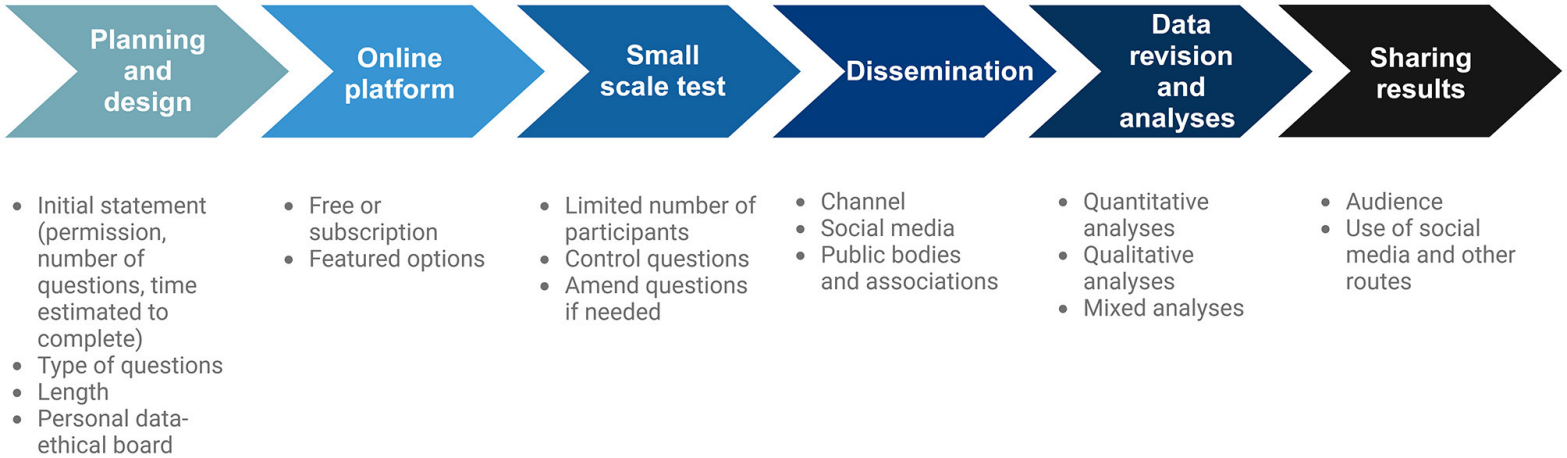
Proposed pipeline for conducting online surveys.

Although there are service providers who specialize in designing and conducting surveys, researchers can directly do both. However, conducting surveys might pose some unexpected challenges that can complicate the execution of the primary objective. Here, we briefly compile some considerations based on our experience of conducting surveys relating to microbiology, specifically in areas relating to the gut microbiota and fermented foods, and the lessons that we have learned to help other researchers successfully and effectively design and conduct surveys (Figure 1).

## 2 Planning and design

The first thing you need to consider is whether or not you are collecting personal data. Personal data include any piece of information that could be used to trace back the information provided to a person, including IP identification. If so, you will have to follow the guidelines for data protection in your country and organization. In the case of Europe, the General Data Protection Regulation (GDPR) provides the framework for these endeavors. Additionally, it is likely that you will have to write and submit documentation, including your fully designed survey, to be approved by a registered ethical board before starting your study (Fink, 2015). It is important to note that, if you decide to make any modifications, you will have to communicate these changes to the ethical board for further approval. If your questions are designed to only obtain demographic information, you are exempt from ethical requirements and do not need to undergo this step.

Designing the survey involves developing the questions according to the information you want to obtain. Communication with the participants is also of key importance. You will have to inform them about the purpose of your survey, how many questions are included, and an estimate of the time required to complete the survey, provide a contact, and explicitly ask the participant whether they want to be a part of the study, before proceeding with the survey questions. You can ask questions with yes/no answers, multiple choice, select the correct answer, fill in the blanks, order the options, etc. The type of question can influence the number of responses that you receive, i.e., a question that requires an extended written or typed response would be less likely to be answered than a yes/no question. Consider providing a choice for “opt out” in the form of “I prefer not to say,” “I don't know,” “Not applicable,” etc. (Penn and Hu, 2021). This will increase the likelihood of receiving responses to all questions. Similarly, provide a space at the end of the survey for people to leave feedback, as many people may wish to express their opinions or provide some additional insight.

Consider including control questions, i.e., if you are particularly interested in acquiring information relating to a specific topic, there is merit in evaluating the participant's knowledge in various ways, as people can respond differently if the question is phrased differently.

Finally, consider the length of your survey. Ideally, a survey should be short and completed in a few minutes (3–5), as the longer the survey, the lower the completion rates (Kost and Rosa da, 2018). For that, it would be useful to include in your initial statement the number of questions and how long it is estimated that the survey can be completed.

## 3 The platform

Traditionally, surveys have been conducted via mail, telephone, or in person, and some studies are still completed using these methods. However, online surveys can be faster, reach more potential participants, and be more cost-effective (Evans and Mathur, 2018).

There are different platforms where you can design and host an online survey according to your needs (Table 1). One free-of-charge option is to use Google Forms. This is a very intuitive platform and offers different question types. The form can be disseminated by simply sharing a link. Many of the platforms that specialize in surveys also provide free-of-charge options. These free options differ with respect to the number of surveys you can conduct, the number of questions you can ask, the number of responses you can collect, etc. Some options, such as Survicate's free plans, allow unlimited surveys and answers. The subscription plans provided by other platforms include featured options, normally including customization, use of different languages, analytical features, and/or unlimited responses. Some of the most commonly used platforms include SurveyMonkey, Qualtrics, and SurveyPlanet. Some institutions may have subscriptions in place that researchers can avail of. Alternatively, if you are conducting a survey after a talk, a seminar, or an outreach activity, such as citizen science, you can consider using mobile applications, such as Socrative, where you can design your questions and receive the answers in real time.

**Table 1:** Detailed free features of frequently used survey platforms.

**Platform**	**Link**	**Free or subscription**	**Number of surveys free version**	**Number of questions free version**	**Number of answers free version**	**Featured options**
Google forms	www.docs.google.com/forms/	Free	Unlimited	Unlimited	Unlimited	-
Jotform	www.jotform.com	Both	5	100	100	More monthly submissions, more questions/surveys, etc.
SurveyMonkey	www.surveymonkey.com	Both	Unlimited	10	100	Languages, analyses, etc.
Qualtrics	www.qualtrics.com	Both	3	30	500	Languages, analyses, unlimited answers, etc.
SurveyPlanet	www.surveyplanet.com	Both	Unlimited	Unlimited	Unlimited	Export results, completion notifications, etc.
Survicate	www.survicate.com	Both	Unlimited	100 across all active surveys	25/month	Mobile, website, languages, analyses, etc.
SoGoSurvey	www.sogolytics.com	Both	Unlimited	Unlimited	200/year	QR code, send reminders, etc.
Zoho Survey	www.zoho.com	Both	Unlimited	10	100	Exports in XLS, SPSS, CSV, more seats, audits, etc.
Typeform	www.typeform.com	Both	Unlimited	10	10/moth	Integration in platforms, different users, subdomains, etc.
Mailchimp	www.mailchimp.com	Both	Unlimited	Unlimited	1,000/month	Templates, more seats, analyses, etc.
Survey builder	www.onlinesurveybuilder.com	Both	5	20	100/survey	Customize, more answers and surveys per month, etc.
Survey sparrow	www.surveysparrow.com	Both	3	10	50/month	Schedule surveys, security, branding, etc.

## 4 The ride test

It is advisable to conduct a small test with a limited number of participants to confirm that the answers are understood and that no final amendments need to be made. One approach to revealing potentially ambiguous questions is to identify responses to control questions that are contradictory; e.g., in one of our tests, participants indicated they knew how to order a gut microbiota analysis, but in our control question, it was clear that they were thinking about a colorectal screening. The question was amended for clarity before going into the definitive release.

## 5 Dissemination

This can frequently be the most challenging stage. *A priori*, it might seem like an easy task to just circulate the survey, but we have observed that it is difficult to reach high levels of completion when randomly circulated by email or social media.

It is important to consider your audience and identify which channels of dissemination are most likely to lead to results where the audience becomes aware of and completes the survey. For example, if you are interested in the general public's opinion on a topic but circulate the survey only through academic channels, the initiative will not be successful.

Social media has become a powerful means via which surveys can be disseminated very widely. Frequently used options include LinkedIn, Twitter, Facebook, and Instagram. Dissemination through the social media accounts of a researcher's institution or other accounts with a large following, including influencers, can still be valuable. Newly created social media accounts dedicated to the completion of the survey can also be of value.

Sharing via email through different public bodies, such as universities, professional associations, and other organizations is another approach that can be considered. However, such organizations will typically only circulate emails on behalf of researchers affiliated with the organization and will likely want to first screen the survey.

## 6 The analyses and representation of the data

Once you have collected all the data, another challenge can be obtaining meaningful information from it. If you have many variables, it can be difficult to find a clear message. Data should be analyzed thoughtfully, and care should be taken when deciding how best to represent the conclusions based on the research objectives and the type of data collected.

There are various approaches to analyse the data, including quantitative, qualitative, and mixed methods. Quantitative analysis involves using statistical techniques to analyse numerical data collected from surveys, which is useful for examining patterns, relationships, and trends in the data. It involves calculating descriptive statistics, such as means, frequencies, and correlations, as well as conducting inferential statistics, such as *t*-tests or regression analyses, to test hypotheses (Nardi, 2018). Quantitative analyses are suitable for examining large datasets and identifying statistical relationships. This approach, for example, can be easily represented by the use of bar or pie charts.

Qualitative analysis involves analyzing non-numerical data, such as open-ended survey responses or interview transcripts, which would involve understanding the meaning and context of participant's responses. Techniques include thematic and content analyses and grounded theory, focused on identifying themes, patterns, and categories in the data (Loehnert, 2010). Qualitative analysis is valuable for exploring participant's perspectives and generating contextual insights. Representing this type of data can be a bit more challenging. An example of how to represent it is the word cloud, whereby the keywords representing ideas that are most repeated will be effectively identified (DePaolo and Wilkinson, 2014).

Mixed methods combine quantitative and qualitative data with the aim of gaining a comprehensive understanding of the data by validating findings and results, as well as providing a more nuanced interpretation. It involves merging datasets, comparing results, or using one method to explain or expand on the findings of another (Driscoll et al., 2007).

## 7 Sharing your results

There are several strategies to communicate the results of your survey, but their effectiveness will depend on the nature of the survey, the target audience, and the desired level of detail and engagement.

If you are planning to share your results with a scientific audience, the most common and appropriate approach would be either a formal peer-reviewed journal or a research report. However, presenting your data to a broader audience for scientific communication would typically involve the use of less technical language and more visual representations. Infographics can be used to present the key findings in a concise and visually appealing manner via the use of charts, graphs, and illustrations (Krum, 2013). Similarly, data dashboards present the information in an interactive way, even providing real-time results and allowing readers to filter and visualize results based on their specific interests (Smith, 2013). A very useful strategy involves the use of summary reports, which condense the key findings and implications in a digestible and accessible way for a broader audience and are used to transfer knowledge to policymakers or media outlets (Mea et al., 2016).

All of these approaches can be further shared in another communication layer using social media and online platforms such as X, Facebook, LinkedIn, or Instagram to reach a broader audience, or in presentations conducted in person to smaller audiences or conducted online for larger public audiences.

## 8 Conclusion

As the importance of scientific communication is increasingly appreciated, surveys are proving to be a valuable tool for gathering data and information and collecting perspectives and opinions that will allow the researcher to gain insight into public perceptions of a given topic and design communication strategies accordingly. Moreover, surveys can be used to assess the effectiveness of scientific communication strategies and interventions and help in the development of more effective communication channels and platforms, ensuring that scientific knowledge reaches the intended audience in a timely and accessible manner. However, the reliability of the results is dependent on careful survey design and the appropriate analyses and interpretation of the data, which will require careful and educated consideration.

## Author contributions

EG-G: Conceptualization, Writing—original draft, Writing—review & editing, Funding acquisition. LW: Writing—review & editing. PC: Conceptualization, Writing—review & editing, Funding acquisition.
